# Research Note: Oral administration with taurine alleviates the weight loss, intestinal atrophy, and redox imbalance induced by post-hatch transportation in yellow-feathered broiler chicks

**DOI:** 10.1016/j.psj.2025.105928

**Published:** 2025-10-01

**Authors:** Wenling Huang, Yang Dai, Leru Deng, Shuhan Pan, Yucheng Yin, Shangcong Wu, Zhitong Fan, Yizheng Hong, Huihua Zhang, Cui Zhu

**Affiliations:** School of Animal Science and Technology, Foshan University, Foshan 528225, China

**Keywords:** Transport stress, Broiler chicks, Taurine, Antioxidant capacity, Intestinal morphology

## Abstract

This study investigated the effects of oral taurine (Tau) administration on the weight loss, plasma biochemical parameters, intestinal integrity, and redox status in yellow-feathered broiler chicks exposed to post-hatch transport stress (TS). A total of 180 newly hatched yellow-feathered broiler chicks were selected and randomly assigned to 5 treatment groups, with 6 replicates each group and 6 chicks each replicate. The broilers were orally administrated with either physiological saline or 1 %, 2 %, and 3 % Tau followed by 5 h post-hatch transportation except the negative control (NC) group. The results showed that TS significantly increased the weight loss, rectal temperature, heart rate, plasma lactate dehydrogenase (LDH) and malondialdehyde (MDA) levels, as well as the crypt depth in duodenum and ileum, while reducing villus height of duodenum, jejunum, and ileum in chicks (*P* < 0.05). However, oral Tau administration alleviated the weight loss induced by TS, and reduced plasma aspartate aminotransferase (AST), alanine aminotransferase (ALT), and LDH activities, and MDA levels in chicks (*P* < 0.05). Additionally, the villus heights (VH) and the VH to crypt depth (CD) ratios in duodenum, jejunum and ileum, as well as plasma glutathione peroxidase (GSH-Px) activity, were significantly increased by oral Tau treatment in yellow-feathered broiler chicks subjected to TS, while the crypt depths in duodenum, jejunum and ileum were decreased by Tau administration (*P* < 0.05). These findings demonstrate that oral Tau administration could mitigate TS-induced weight loss, intestinal atrophy and redox imbalance in yellow-feathered broiler chicks by enhancing intestinal integrity and improving antioxidant capacity. The optimal dose of Tau for newly-hatched chicks prior to TS is 2 % (equivalent to 575 mg/kg body weight) when administrated orally.

## Introduction

Chick transportation from hatcheries to growing facilities is a critical but high-risk phase in modern poultry production systems. During transit, chicks face multifactorial stressors including thermal fluctuations, feed deprivation, and physical vibration that cause physiological disturbances resulting in an increase of mortality and body weight loss (Yerpes et al., 2021). These challenges not only compromise animal welfare, but also predispose chicks to subsequent production impairments, which lead to great economic losses in global broiler industry. Therefore, finding effective nutritional strategies to alleviate the negative effects of transport stress (TS) has gained more attention based on the above urgent needs.

Taurine (Tau), a sulfur-containing β-amino acid with potent antioxidant and anti-inflammatory properties, has emerged as a promising feed additive in livestock production. Poultry studies have demonstrated that dietary Tau supplementation could improve antioxidant capacity in heat-stressed broilers (Wang et al., 2022), reduce the cold stress-induced hepatic oxidative injury (Lyu et al., 2022), and attenuate lipopolysaccharide (LPS)-induced intestinal inflammation (Han et al., 2020). Furthermore, previous research in weaned piglets has shown that dietary Tau supplementation significantly enhanced growth performance and intestinal integrity (Chen et al., 2024). Notably, another study in ruminant also validated the efficacy of Tau supplement in alleviating TS responses (Wang et al., 2024), suggesting its potential for poultry applications. Importantly, newly hatched chicks with immature digestive and antioxidant systems, coupled with prolonged feed deprivation during transit, are particularly vulnerable to TS-induced damage (Yerpes et al., 2021). Despite Tau's documented protective effects against various stressors, its possible role in alleviating TS-mediated intestinal injury and oxidative imbalance in neonatal chicks remains unexplored.

Thus, this study evaluated the effects of oral Tau administration on body weight (BW) loss, intestinal morphology, and redox status in yellow-feathered broiler chicks subjected to post-hatch transportation. We hypothesized that Tau would mitigate TS-induced intestinal damage by enhancing antioxidant defenses and maintaining gut integrity, aiming to provide a practical nutritional strategy to improve chick welfare and production efficiency during this critical phase.

## Materials and methods

### Animal design and treatments

All experimental methods of animals were performed according to institutional animal care and use committee guidelines under protocols approved by the Institutional Animal Care and Use Committee of Foshan University (FOSU2022004). A total of 180 female newly hatched chicks with average initial BW of 34.8 ± 0.1 g were obtained from a commercial farm (Foshan, China) and assigned to 5 groups, including the negative control (NC) group, TS group, or 1 %, 2 %, and 3 % Tau groups. Each group had 6 replicates (*n* = 6), with 6 chicks each replicate. The newly-hatched broiler chicks were orally administrated with 1 mL of either physiological saline or 1 %, 2 %, and 3 % Tau solution dissolved in physiological saline. Given the average initial body weight, the Tau doses for the latter three groups were equivalent to 287 mg/kg BW, 575 mg/kg BW, and 862 mg/kg BW, respectively. The broiler chicks in the NC group were placed in the box (62 cm × 47 cm × 16 cm) without transportation, while the broiler chicks in other four groups were placed in the same sized-boxes and then subjected to post-hatch transportation to induce TS model in a 5-h journey at 70 km/h without abrupt braking or acceleration during the transport trip. The temperature in the transport vehicle was maintained at 29°C, while the relative humidity was maintained at 68 %.

### Sample collection

After 5-h post-hatch transport, the rectal temperature of chicks was recorded by inserting a thermometer probe approximately 2-3 cm into the rectum and holding it steady for 15 s. Moreover, the heart rate of chicks was recorded by a veterinary technician counting beats per minute (bpm). Blood samples (*n* = 6) were then collected from the wing vein using heparinized tubes and centrifuged at 1,006 g, 4°C for 10 min. The plasma samples were stored at −80°C for subsequent analysis of biochemical parameters and antioxidant capacity indicators. After blood sampling, the yellow-feathered broiler chicks were euthanized for sample collection under ethical guidelines. Segments of the duodenum, jejunum, and ileum of approximately 10 cm were excised and rinsed with ice-cold PBS to remove digesta, and then fixed in 10 % neutral buffered formalin for histological analyses.

### Determinations of intestinal morphology

Intestinal morphology was assessed according to the previous method (He et al., 2019). Briefly, segments of the duodenum, jejunum, and ileum were collected, fixed in 10 % neutral buffered formalin, and embedded in paraffin. Tissue sections (5 μm) were stained with hematoxylin and eosin (H&E). Villus height (VH), crypt depth (CD), and their ratio (VH/CD) of ten intact villi and crypts per sample were measured using Image J software (NIH, USA) under a light microscope (400× magnification).

### Determinations of plasma biochemical parameters

Plasma concentrations of total protein (TP), albumin (ALB), triglycerides (TG), total cholesterol (TC), glucose (GLU), and uric acid (UA), as well as the activities of lactate dehydrogenase (LDH), aspartate aminotransferase (AST), and alanine aminotransferase (ALT) were determined using the commercial assay kits (Nanjing Jiancheng Bioengineering Institute, China) following the manufacturer’s protocols.

### Determinations of plasma antioxidant capacity

Plasma antioxidant capacity parameters including superoxide dismutase (SOD) activity, glutathione peroxidase (GSH-Px) activity, malondialdehyde (MDA) content, and total antioxidant capacity (T-AOC) were determined using the commercial kits (Nanjing Jiancheng Bioengineering Institute, China), according to the manufacturer's instructions.

### Statistical analyses

The data were analyzed using student’s t-test for the results of rectal temperature and heart rate, or using one-way ANOVA for other results followed by Duncan's multiple comparison test in SPSS software (version 26.0; IBM Corp., Armonk, NY). Data are presented as means ± standard error (SE). Differences were considered statistically significant at *P* < 0.05.

## Results and discussion

### Weight loss, rectal temperature, and heart rate

**Fig. 1:**
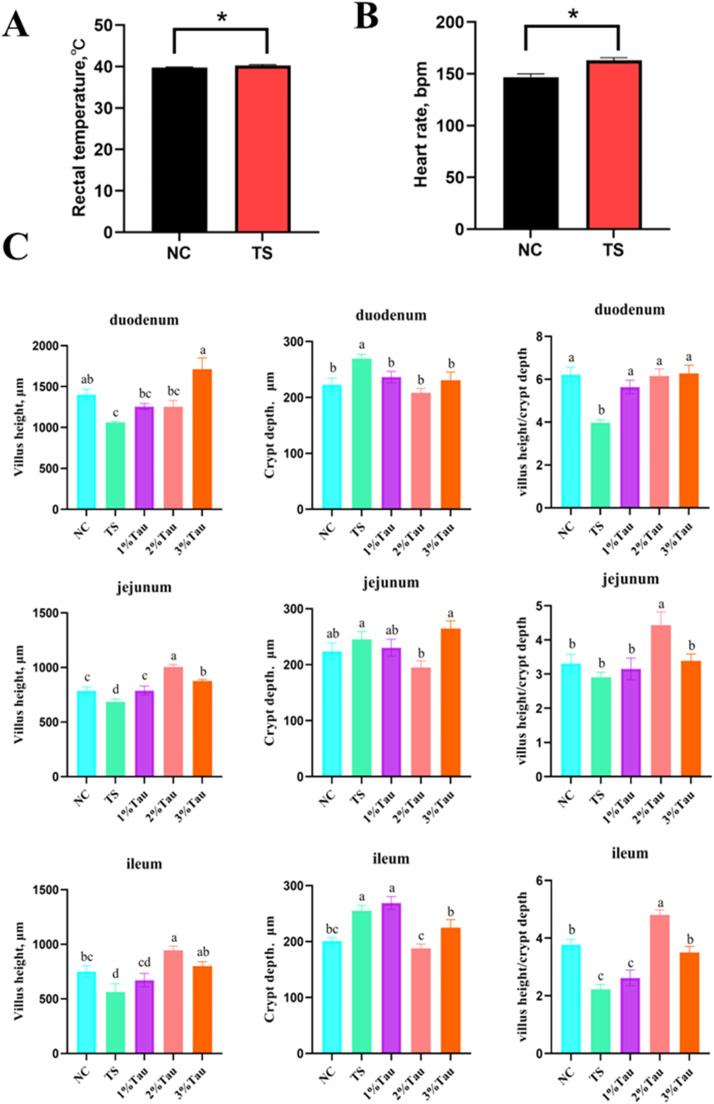
Effect of transport stress on rectal temperature and heart rate, and oral administration with taurine on intestinal morphology in yellow-feathered broiler chicks subjected to post-hatch transportation for 5 h. (A) Rectal temperature. (B) Heart rate. (C) The villus height, crypt depth, and villus height to crypt depth ratio of the duodenum, jejunum, and ileum. ^a-c^The means with no common superscripts within each row are significantly different (*P* < 0.05). Abbreviations: NC, negative control group; TS, transport stress; Tau, taurine.

The post-transport BW was significantly decreased, and the weight loss (Table 1), rectal temperature (Fig. 1A), and heart rate (Fig. 1B) after 5 h-transportation were significantly increased in the broiler chicks of TS group when compared to the negative control group (NC) (*P* < 0.05), indicating the effective establishment of TS model in current study. This observed increases in rectal temperature and heart rate might be primarily due to the activation of the sympathetic nervous system and the release of stress-related hormones, which could elevate metabolic rate and cardiac output following TS. Consistently, Yerpes et al. (2021) has shown that transportation of day-old chicks increased weight loss during transport under commercial conditions, with journey duration negatively affecting the body weight of chicks. However, oral administration with 1 %, 2 % or 3 % Tau significantly attenuated the weight loss induced by TS in broiler chicks (*P* < 0.05). To our knowledge, this was the first report demonstrating the protective role of Tau in restoring the weight loss in yellow-feathered broiler chicks followed by post-hatch transportation. The result of current study was consistent with previous study that increasing Tau supplementation levels in the diet reduced post-transport weight loss and weight loss percent, and alleviated the stress responses in yaks (Wang et al., 2024).

**Table 1:** Effect of oral administration with taurine on weight loss, plasma biochemical parameters and antioxidant capacity in yellow-feathered broiler chicks subjected to post-hatch transportation for 5 h.

Item	NC	TS	1 %Tau	2 %Tau	3 %Tau	*P* value
**Body weight, g**						
Pre-transport	34.8 ± 0.1	34.8 ± 0.1	34.8 ± 0.1	34.8 ± 0.1	35.0 ± 0.1	0.053
Post-transport	34.4 ± 0.1^ab^	34.1 ± 0.1^c^	34.2 ± 0.1^bc^	34.3 ± 0.1^abc^	34.5 ± 0.1^a^	0.004
Weight loss	0.38±0.02^c^	0.61±0.02^a^	0.56±0.01^b^	0.53±0.01^b^	0.53±0.02^b^	<0.001
**Plasma biochemical parameters**						
TP, ug/mL	1354±69	1206±32	1285±59	1150±61	1215±51	0.151
ALB, g/L	0.50±0.02	0.49±0.02	0.48±0.02	0.48±0.02	0.48±0.03	0.942
TG, mmol/L	0.34±0.04	0.35±0.01	0.34±0.03	0.39±0.03	0.37±0.02	0.721
TC, mmol/L	7.1 ± 0.4	7.5 ± 0.9	6.6 ± 0.8	7.9 ± 0.9	7.1 ± 0.7	0.772
GLU, mmol/L	10.2 ± 0.3	9.8 ± 0.4	9.9 ± 0.3	9.6 ± 0.2	10.5 ± 0.6	0.511
UA, μmol/L	235±37	305±12	198±12	265±41	221±26	0.062
LDH, U/L	12198±413^b^	13757±491^a^	12047±385^b^	13275±479^ab^	12160±311^b^	0.027
AST, U/L	6.3 ± 0.8^abc^	9.6 ± 1.3^a^	4.0 ± 1.0^c^	8.7 ± 1.7^ab^	5.1 ± 1.3^bc^	0.028
ALT, U/L	3.4 ± 0.7^ab^	5.1 ± 1.3^a^	1.7 ± 0.6^bc^	0.8 ± 0.2^c^	1.9 ± 0.7^bc^	0.003
**Plasma antioxidant capacity**						
MDA, nmol/mL	5.2 ± 0.3^bc^	6.7 ± 0.4^a^	4.0 ± 0.3^c^	5.1 ± 0.2^bc^	6.2 ± 0.6^ab^	0.003
T-AOC, mM	0.53±0.05	0.47±0.05	0.62±0.03	0.66±0.1	0.62±0.08	0.255
GSH-Px, U/mL	1842±29^ab^	1677±12^b^	1783±43^b^	1966±76^a^	1993±50^a^	<0.001
SOD, U/mL	83.2 ± 1.3	77.7 ± 4.2	83.7 ± 1.1	84.3 ± 3.5	82.7 ± 3.3	0.787

^a-c^The means with no common superscripts within each row are significantly different (*P* < 0.05).

Abbreviations: NC, negative control; TS, transport stress; Tau, taurine; TP, total protein; ALB, albumin; TG, triglycerides; TC, total cholesterol; GLU, glucose; UA, uric acid; LDH, lactate dehydrogenase; AST, aspartate aminotransferase; ALT, alanine aminotransferase; malondialdehyde (MDA); total antioxidant capacity (T-AOC); glutathione peroxidase (GSH-Px), superoxide dismutase (SOD).

### Plasma biochemical parameters

Compared with the NC group, TS significantly increased the plasma AST, ALT and LDH levels in yellow-feathered broiler chicks (*P* < 0.05) (Table 1). Consistently, previous study in yaks also found that the plasma concentrations of LDH, LPS, creatine kinase, and cortisol were significantly increased by TS (Wang et al., 2024). However, compared to the TS group, oral administration with 2 % and 3 % Tau significantly reduced plasma LDH, ALT and AST activities, with no significant differences in plasma TP, ALB, TG, TC, GLU, UA levels of yellow-feathered broiler chicks among different treatments (*P* > 0.05) (Table 1). Similarly, previous study has shown that the increases in serum ALT and AST activities induced by LPS challenge were reversed by Tau supplementation (Han et al., 2020). Moreover, adding 2 g/L Tau into drinking water significantly increased the total serum TP content, and lowered serum UA levels and serum activities of creatine kinase, LDH, AST, and ALT in broilers under heat stress (Yang et al., 2022). These results indicated that Tau could modulate the metabolic function of animals under stress condition during transportation.

### Plasma antioxidant capacity

Compared with the control group, TS significantly increased the levels of MDA in the plasma (*P* < 0.05) (Table 1), indicating that TS might induces oxidative stress by altering the redox balance of yellow feathered broiler chicks. This was in accordance with previous study that severe oxidative stress was observed in yaks subjected to TS (Wang et al., 2024). However, compared with TS group, oral administration with 1 % or 2 % Tau significantly reduced the plasma MDA levels in yellow-feathered broiler chicks (*P* < 0.05), with no significant difference in plasma T-AOC and SOD activities among the groups (*P* > 0.05) (Table 1). Similarly, serum MDA concentration was significantly decreased by 4 g/kg Tau supplementation in the diets of Cobb chicks for 1, 2 and 3 weeks (Huang et al., 2014). In addition, the present study found that oral administration with 2 % or 3 % Tau significantly increased the plasma GSH-Px activity in yellow-feathered broiler chicks (*P* < 0.05) (Table 1). This was in consistent with previous study that dietary Tau treatment could alleviate the hepatic oxidative stress, by lowering MDA content and increasing glutathione and GSH-Px activity in LPS-challenged Arbor Acres broilers (Han et al., 2020). Moreover, adding 1 % Tau in drinking water significantly increased the activities of GSH-Px while reducing MDA concentration in broilers under low temperature (Lyu et al., 2022). The current results indicated that Tau could effectively counteract oxidative damage induced by TS in yellow-feather broiler chicks, which was in aligned with a previous study reporting the mitigating effect of Tau supplement in yaks followed by TS exposure via improvement of antioxidant capacity (Wang et al., 2024). This may be explained by the fact that Tau functions as fundamental mediator of homeostasis to protect against various oxidative stress due to its ability to sustain normal electron transport chain, maintain glutathione stores, upregulate anti‑oxidant responses, increase membrane stability, eliminate inflammation and prevent calcium accumulation (Baliou et al., 2021). Notably, the discrepancy of the dose-dependent effects of Tau in these observations indicate that 2 % Tau may be the optimal dose for mitigating lipid peroxidation in this specific model.

### Intestinal morphology

As indicated in Fig. 1C, TS significantly reduced the villus height of duodenum, jejunum, and ileum and the villus to crypt ratio of duodenum and ileum, while increasing crypt depth of the duodenum and ileum in yellow-feathered broiler chicks when compared to the control group (*P* < 0.05). This was in accordance with the aforementioned TS-induced oxidative imbalance leading to intestinal mucosal damage of chicks. Compared with TS Group, oral administration with 2 % Tau prior to TS significantly increased villus height and the villus to crypt ratio in jejunum and ileum, while decreasing the crypt depth in duodenum, jejunum, and ileum (*P* < 0.05). Consistently, previous study has showed that dietary Tau supplementation effectively mitigated the impairment of jejunal morphology by increasing in villus height as well as the villus height to crypt depth ratio in broilers subjected to chronic heat stress (He et al., 2019). These results confirm that Tau treatment plays a crucial role in protecting the intestinal mucosa from oxidative damage under stressors during transportation. The alleviation of TS-induced intestinal atrophy by oral Tau administration in chicks may be attributed to its multifaceted roles, including osmoregulation, bile acid conjugation, membrane stabilization, and potent antioxidant and anti-inflammatory activities (Baliou et al., 2021). However, whether the mechanism of Tau on protecting gut integrity of neonatal chicks subjected to TS involves the regulation of yolk passage, gut mobility and digestive secretions, or mitochondrial function requires further investigations. The present findings could provide scientific guidance for developing Tau administration as effective nutritional strategies to alleviate TS in poultry production for advancing animal welfare and farming efficiency.

## Conclusion

The results of present study demonstrated that oral administration with Tau effectively mitigates the weight loss, intestinal atrophy, and oxidative imbalance caused by TS in yellow-feathered broiler chicks by enhancing intestinal integrity and improving antioxidant capacity. Tau may represent a valuable nutritional strategy for alleviating the negative impacts of TS on neonatal chicks during commercial transportation. For newly-hatched chicks prior to TS, the optimal Tau administration would be an oral dose of 2 % (equivalent to 575 mg/kg BW). Future researches are needed for optimizing Tau dosage and exploring its synergistic effects with other functional additives to maximize its benefits in poultry production.

## Acknowledgments

This study was supported by the financial support provided by the Guangdong Province Poultry Industry System Project (2024CXTD20) and the Special Project for Key Areas of Guangdong Provincial Department of Education, China (2024ZDZX2092).

## CRediT authorship contribution statement

**Wenling Huang:** Writing – original draft, Methodology, Formal analysis, Data curation. **Yang Dai:** Resources, Methodology, Investigation, Formal analysis. **Leru Deng:** Writing – original draft, Methodology, Formal analysis. **Shuhan Pan:** Methodology, Formal analysis, Data curation. **Yucheng Yin:** Investigation, Formal analysis. **Shangcong Wu:** Formal analysis. **Zhitong Fan:** Investigation. **Yizheng Hong:** Investigation. **Huihua Zhang:** Validation, Resources. **Cui Zhu:** Writing – review & editing, Writing – original draft, Supervision, Project administration, Methodology, Formal analysis, Conceptualization.

## Disclosures

We declare that we have no financial and personal relationships with other people or organizations that can inappropriately influence our work, there is no professional or other personal interest of any nature or kind in any product, service and/or company that could be construed as influencing the content of this paper.
